# Sphingolipid Synthesis Inhibition by Myriocin Administration Enhances Lipid Consumption and Ameliorates Lipid Response to Myocardial Ischemia Reperfusion Injury

**DOI:** 10.3389/fphys.2019.00986

**Published:** 2019-08-09

**Authors:** Fabiola Bonezzi, Marco Piccoli, Michele Dei Cas, Rita Paroni, Alessandra Mingione, Michelle M. Monasky, Anna Caretti, Chiara Riganti, Riccardo Ghidoni, Carlo Pappone, Luigi Anastasia, Paola Signorelli

**Affiliations:** ^1^Stem Cells for Tissue Engineering Laboratory, IRCCS Policlinico San Donato, Milan, Italy; ^2^Clinical Biochemistry and Mass Spectrometry Laboratory, Health Sciences Department, University of Milan, Milan, Italy; ^3^Biochemistry and Molecular Biology Laboratory, Health Sciences Department, University of Milan, Milan, Italy; ^4^Arrhythmology Department, IRCCS Policlinico San Donato, Milan, Italy; ^5^Cell Biochemistry Laboratory, Oncology Department, and Interdepartmental Research Center for Molecular Biotechnology, University of Turin, Turin, Italy; ^6^Department of Biomedical Sciences for Health, University of Milan, Milan, Italy

**Keywords:** sphingolipids, ceramide, myriocin, ischemia, reperfusion, metabolism

## Abstract

Myocardial infarct requires prompt thrombolytic therapy or primary percutaneous coronary intervention to limit the extent of necrosis, but reperfusion creates additional damage. Along with reperfusion, a maladaptive remodeling phase might occur and it is often associated with inflammation, oxidative stress, as well as a reduced ability to recover metabolism homeostasis. Infarcted individuals can exhibit reduced lipid turnover and their accumulation in cardiomyocytes, which is linked to a deregulation of peroxisome proliferator activated receptors (PPARs), controlling fatty acids metabolism, energy production, and the anti-inflammatory response. We previously demonstrated that Myriocin can be effectively used as post-conditioning therapeutic to limit ischemia/reperfusion-induced inflammation, oxidative stress, and infarct size, in a murine model. In this follow-up study, we demonstrate that Myriocin has a critical regulatory role in cardiac remodeling and energy production, by up-regulating the transcriptional factor EB, PPARs nuclear receptors and genes involved in fatty acids metabolism, such as VLDL receptor, Fatp1, CD36, Fabp3, Cpts, and mitochondrial FA dehydrogenases. The overall effects are represented by an increased β–oxidation, together with an improved electron transport chain and energy production. The potent immunomodulatory and metabolism regulatory effects of Myriocin elicit the molecule as a promising pharmacological tool for post-conditioning therapy of myocardial ischemia/reperfusion injury.

## Introduction

Myocardium infarct and heart failure are leading causes of morbidity and mortality in developed countries. When ischemia occurs, immediate thrombolytic therapy or primary percutaneous coronary intervention is required to limit the extent of necrosis (Frank et al., 2012). On the other hand, inevitably, reperfusion induces additive injury, estimated to contribute up to 50% of the infarct lesion, ultimately causing functional impairment and arrhythmia in the post-infarct phase, when damages extend to ventricular dilation, mitral valve regurgitation, loss of contractile force, ionic unbalance, and biochemical, neuroendocrine, and energy metabolism changes. Such a vicious cycle, named “cardiac remodeling,” afflicts approximately 30% of infarcted patients (Picard et al., 1990; Flachskampf et al., 2011; Konstam et al., 2011; Frank et al., 2012; Curley et al., 2018). In the normal heart, fatty acid (FA) and glucose metabolism are both required and tightly regulated, although nearly 70% of cardiac ATP derives from FA oxidation (Park and Goldberg, 2012). Upon ischemia, the myocardium immediately shuts off oxygen-consuming processes, and it temporarily relies on anaerobic glycolysis. This process, while sparing oxygen, results in poor levels of ATP and reduces cell pH because of lactate accumulation. Once blood flow is restored, mitochondrial oxidative phosphorylation is recovered, and aerobic glycolysis and FA oxidation activity are restored. In this recovery phase, depending on the severity of the ischemia/reperfusion (I/R) injury, the overall oxidative metabolism may be depressed, thus reducing energy fueling and contributing to maladaptive myocardial remodeling, which leads to hypertrophy and heart failure. In this latter condition, the myocardium switches to a fetal-like profile that includes down-regulation of myosin heavy chain (MHC) -α and up-regulation of its fetal isoform β (Lowes et al., 1997), associated with contractile dysfunction, low lipid/high glucose metabolic consumption, poor ATP production (Qanud et al., 2008; Karwi et al., 2018) and lipid overload (Schulze et al., 2016). Any given post-conditioning therapy is aimed at limiting I/R additive damages. Unfortunately, anti-inflammatory post-conditioning therapies failed in human trials, indicating that a regulated inflammatory response and its resolution are necessary for functional tissue recovery in the post-infarction phase (Huang and Frangogiannis, 2018).

Recently, a pathological role of lipids metabolism deregulation had been proposed in tissue remodeling. Cardiac adipose tissue surrounds the heart (epicardial and pericardial fat), providing mechanical support. Moreover, myocardium buffers the excess of circulating free FA by metabolic consumption, contributing to total body lipid homeostasis (Antonopoulos and Antoniades, 2017). Advanced morphological and functional assessment enabled the identification of metabolic changes in lipid metabolism in the infarcted myocardium (Wong et al., 2017). Infarcted individuals exhibit an accumulation of fat in the left ventricle that reaches up to 60% throughout the ventricle (from 20% of the basal area only in healthy myocardium) (Pouliopoulos et al., 2013; Sasaki et al., 2015). Increased intra-myocardial fat was related to altered electrical activity (Iozzo, 2011; Chhabra and Gurukripa Kowlgi, 2015; Samanta et al., 2016; Wong et al., 2017) and a direct association was demonstrated between myocardial dysfunction (Schulze, 2009) and fat replacement of fibrous tissue within ischemia-produced scar (Pouliopoulos et al., 2013; Sasaki et al., 2015).

Reduced lipid turnover and accumulation is associated with altered expression and function of peroxisome proliferator activated receptors (PPARs) family of nuclear receptors, regulating FAs metabolism, energy production and anti-inflammatory response (Ravingerova et al., 2011). The three isoforms, PPAR-α, PPAR-β/δ and PPAR-γ are all expressed in the myocardium, although to a different extent, and the α and β/δ subtypes are down-regulated in pathological myocardial inflammation, fetal metabolic profile switch and hypertrophy (Liang et al., 2003; Smeets et al., 2008a,b). Activation of PPAR-γ in infarcted myocardium improved contractile recovery and/or reduced infarct size after ischemia and reperfusion (Yue et al., 2005; Geng et al., 2006; Cao et al., 2007; Liu et al., 2009; Yasuda et al., 2009). Interestingly, PPAR-α and PPAR-γ synthetic agonists are in use as hypolipidemic and antidiabetic drugs and have been reported to protect the heart against ischemia/reperfusion injury (Ravingerova et al., 2011). Ectopic lipid accumulation in the myocardium, and in other tissues, harbors lipid droplets formation, a marker of lipid overload, whose role is to store and coordinate lipid intracellular transfer use and oxidation, by the action of specialized proteins such as lipases and perilipins (Goldberg et al., 2018). Lipid droplets accumulation in cardiac left ventricle can be envisaged in fasting (Trent et al., 2014) or hypoxia (Mazzali et al., 2015). Different species are known to give rise to lipids accrual, with either structural, energetic or signaling roles. Triacylglycerol, cholesterol and retinyl ester lipids, ether lipids as well as lipotoxins, such as free cholesterols, diacylglycerols, and ceramides, are found associated with lipid droplets (Goldberg et al., 2018).

Ceramide, a sphingoid base containing molecule, is an important intermediate forming the membrane components sphingomyelins and glycosphingolipids. Its synthesis, initiated by serine palmitoyl transferase (SPT), is tightly regulated with the synthesis rate of other lipids such as cholesterol and phosphatidylcholine (Ghidoni et al., 2015). Moreover, ceramide cellular content increases significantly upon stress, affecting cell cycle, inflammation, and survival. Ceramide accumulation is also related to reduced PPARs activity (Gao et al., 2015). Ceramide increase and signaling is a pathological hallmark in a variety of diseases, and it is a clinically recognized target in cancer, neurodegenerative, inflammatory and cardiovascular diseases therapies (Maceyka and Spiegel, 2014; Di Pardo and Maglione, 2018; He and Schuchman, 2018).

Myriocin, which is a specific inhibitor of SPT and of ceramide *de novo* synthesis, has been extensively used *in vitro* and *in vivo* in several animal models (Campisi et al., 2017). Myriocin was demonstrated to reduce myocardial ceramide and its related insulin resistance (Ussher et al., 2012; Hodson et al., 2015), as well as oxidative stress (Tippetts et al., 2014; Nelson et al., 2015). Myriocin reduced cardiac dilation and improved contractile function in a murine model of cardiomyopathy (Park et al., 2008). We recently demonstrated that Myriocin post-conditioning by intra-ventricular injection of Myriocin-loaded nanoparticles significantly reduces I/R lesion and inflammation in the first hours after reperfusion (Reforgiato et al., 2016) (patent number: US 9925160 B1). Moreover, it has been shown that dietary administration of Myriocin in the post-infarction phase ameliorates myocardial remodeling and function (Ji et al., 2017). The present data demonstrate that this molecule is able to modulate cell metabolism and to promote the activation of genes involved in the control of inflammation and of lipid consumption to produce energy.

## Materials and Methods

### Solid Lipid Nanocarriers (SLN)–Myriocin Preparation

Solid lipid nanocarriers (SLN) loaded with myriocin (SLN/myr, 1 mM) were prepared as previously described (Caretti et al., 2014) by Nanovector S.r.l., Italy, and stored at −80°C until use. SLN/myr stock was diluted 1:4 in 0.9% NaCl sterile solution.

### Ethics Statement

Animal studies were conducted in accordance with the Guide for the Care and Use of Laboratory Animals (National Institutes of Health, Publication No. 85–23, revised 1996) and with the ARRIVE guidelines and adhered strictly to the Italian Ministry of Health guidelines for the use and care of experimental animals. Authorization number 361/2017-PR by the Italian Institute of Health (Istituto Superiore della Sanità).

### Left Anterior Descending (LAD) Coronary Ligature

We used male C57BL/6N mice (Charles River Laboratories Italia, Italy), 8–10 weeks old. Animals were anesthetized by intraperitoneal injection of Medetomidine, 0.5 mg/Kg (Orion Pharma S.r.l.) and Ketamine, 100 mg/Kg (Merial), diluted in saline solution. When completely unconscious, mice were ventilated through an endotracheal tube at 200 μL of tidal volume with a respiration rate of 110 breaths/min. Mice were placed on a heating pad at 37°C to maintain a constant body temperature and their chests were opened by thoracotomy between the second and the third rib to expose the left ventricle. Once identified and located the LAD coronary, a 7–0 silk suture was passed under the coronary vessel, 2 mm below the tip of the left auricle. Then, the suture was tied with two knots over a 5 mm long piece of polyethylene tubing to simulate ischemia. To confirm the occlusion of the LAD a paler color in the anterior wall of the LV appeared. After 30 min of ischemia, the tubing was removed to simulate the reperfusion phase (Reforgiato et al., 2016). Groups ranged from five to seven animals each. In the treated group, mice were intra-ventricularly injected, right after the beginning of reperfusion, with 20 μL of SLN/myr, while empty SLN was used in the control group. Mice belonging to the sham group were subjected to the same surgical procedure, with the exception of LAD ligation. The silk suture was left *in loco* for the staining procedure. Animals were reawakened by sub-cutaneous injection of Atipamezole, 5 mg/kg (Orion Pharma S.r.l), and followed for 24 h or 96 h. A sub-cutaneous dose of buprenorphine solution, 0.05 mg/kg (Dechra Pharmaceuticals) was injected immediately after the surgery and, when necessary, every 8–12 h.

### Double-Staining and Quantitative Image Analysis of Heart Sections

Double staining was performed as previously described (Reforgiato et al., 2016) to discriminate surviving cells within the area-at-risk from the dead cells of the infarct area. Briefly, at the end of reperfusion, the LAD coronary was re-occluded by tightening the knot around the vessel and 100 μl of 4% (w/v) Evans blue solution was injected into the apex of the heart to identify the ischemic area by dye exclusion. Then, each heart was weighted and cut into 1 mm thick slices, which were incubated in 1% triphenyl tetrazolium chloride (TTC) (Sigma-Aldrich, United States) in sodium phosphate buffer for 5 min to counterstain surviving cells in the area at risk. The extent of stained and unstained areas was calculated for each slice from digital images using ImageJ software. The area at-risk was expressed as the percentage of total area (the entire surface of each section) whereas the infarct area was expressed as the percentage of the area-at-risk. Percentages were calculated as the mean of all section of one heart. When this imaging procedure was concluded, samples were processed for RNA and lipids extraction.

### Hypertrophy Determination

After 96 h from the LAD occlusion or sham operation, the anesthetized animals and the collected hearts were weighed. Heart weight to body weight ratio (HW/BW; mg/g), a common and simple indicator of cardiac hypertrophy (Chiu et al., 2001; Sugden et al., 2008; Del Olmo-Turrubiarte et al., 2015) was calculated.

### Ceramide Quantitation

The at-risk and infarcted areas and tissue samples collected from sham animals were separated by dissection, weighed and homogenized as previously described (Caretti et al., 2014). The content of dihydroceramide and ceramide species was measured by liquid chromatography-mass spectrometry (LC-MS/MS) following the previously published method (Reforgiato et al., 2016). Sphingolipids species content was normalized to tissue weight, expressed in grams.

### qRT-PCR

Total RNA was isolated from both the myocardial at-risk area and tissue samples collected from sham animals, according to the manufacturer’s instructions (Promega). 1 μg of purified RNA was reverse transcribed with the iScript^TM^ cDNA synthesis kit (Bio-Rad), and the obtained cDNA was stored at −20°C. The amplification of target genes was performed using the GoTaq qPCR Master Mix (Promega) and ROX as reference dye. Amplified genes included: α-myosin heavy chain (*MYH6*), β-myosin heavy chain (*MYH7*), for the hypertrophic analysis; PGC-1α (*PPARGC1A*), PPAR-α (*PPARA*), PPAR-γ (*PPARG*), PPAR-β/δ (*PPARD*) and TFEB (*TFEB*) for the analysis of transcriptional factors; P62 (*SQSTM1*) for the analysis of TFEB activity; VLDLR (*VLDLR*), FATP1 (*SLC27A1*), h-FABP (*FABP3*), CD36 (*CD36*), CPT-1a (*CPT1A*), CPT-1b (*CPT1B*), UCP3 (*UCP3*) for the study of FA transporters; MCAD (*ACADM*), LCAD (*ACADL*), HSL (*LIPE*) for the evaluation of the FA metabolism enzymes; PLIN2 (*PLIN2*), PLIN4 (*PLIN4*), PLIN5 (*PLIN5*) for the analysis of the perilipins involvement in I/R injury. Relative mRNA expression of target genes was normalized to the endogenous RPLI control gene and represented as fold change versus control, calculated by the comparative CT method (ΔΔCT Method). All the primer sequences are reported in the Supplementary Table S1.

### *Ex vivo* Enzymatic Activities

Left ventricular samples were used for mitochondria extraction from tissue homogenates and measurement of oxidative phosphorylation (OXPHOS) (Buondonno et al., 2016) and ATP concentration were performed as previously described (Campia et al., 2009). The rate of reduction of cytochrome c, an index of the electron transport between Complex I–III and OXPHOS rate, was measured spectrophotometrically on 10 μL of non-sonicated mitochondrial samples. The results were expressed as nmoles cyt c reduced/min/mg mitochondrial proteins. FAO analysis was performed as previously reported (Capello et al., 2016), by radiolabeling for 2 h tissue homogenates with 2 μCi [1−^14^C] palmitic acid (3.3 mCi/mmol, PerkinElmer, Waltham, MA, United States) and measuring the labeled acid soluble metabolites (ASM), as products of FAO, by liquid scintillation. The results were expressed as pmoles ^14^C-ASM/h/mg proteins.

### Statistical Analysis

For the animal experiments, five to seven mice were used for each group; all the further anatomical and biochemical analyses were performed on the tissues collected from these groups. Graphs were elaborated using the GraphPad Prism 7 software. Data are expressed as mean ± SD calculated from experimental replicates. Data significance was evaluated by unpaired Student’s *T*-test (significant when *p*-value < 0.05).

## Results

### Single Intra-Myocardial Injection of SLN/myr Lowers Myocardial Ceramide Content, Reducing Infarct Size and Hypertrophy

**FIGURE 1:**
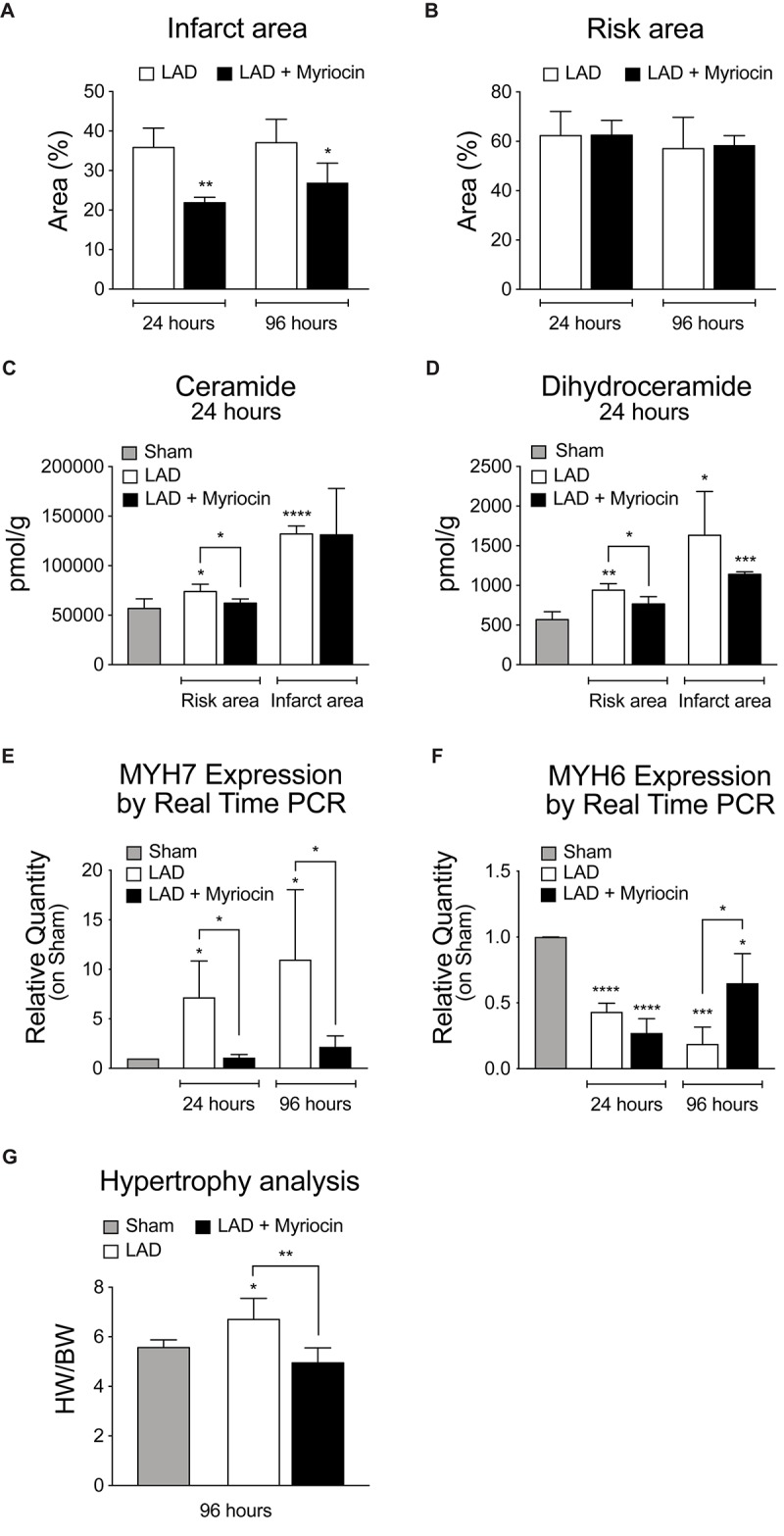
Analysis of SLN/myr treatment effects on infarct areas, sphingolipid content and hypertrophy. **(A)** analysis of the infarct area (expressed as percentage on at-risk area, on the left) and **(B)** of the area at-risk (expressed as percentage on total tissue, on the right) in infarcted myocardium, 24 h and 96 h after surgery. **(C)** LCMS measurement of ceramides and **(D)** dihydroceramides species in infarct and at-risk area, 24 h and 96 h after surgery. qRT-PCR analysis of **(E)** β-myosin heavy chain (MYH7) and **(F)** α-myosin heavy chain (MYH6), 24 h and 96 h after surgery, expressed as relative quantity versus sham group. **(G)** measurement of myocardial hypertrophy, 96 h after surgery, calculated as ratio between heart weight on body weight. All data are expressed as mean ± SD. Statistical significance refers to I/R and SLN/myr treated I/R groups as compared to sham animals. The comparison between I/R and SLN/myr treated I/R groups is indicated by the connecting line (^*^p < 0.05; ^∗∗^*p* < 0.01; ^∗∗∗^*p* < 0.001; ^****^p < 0.0001). Sham animals: gray bar; I/R group: white bar; SLN/myr treated I/R group: black bar.

We performed intra-ventricular injection of SLN/myr at the start of reperfusion (post-conditioning) after left ventricle induced ischemia (LAD, 30 min occlusion). We observed that the SLN/myr post-conditioning significantly decreased the percentage of infarct area (21.87% ± 0.78 at 24 h and 26.88% ± 2.23 at 96 h), as compared to the untreated animals (respectively, 35.85% ± 2.44 and 37.05% ± 2.97), up to 96 h after I/R (Figure 1A). The size of the area-at-risk was not affected by the treatment (Figure 1B), indicating a consistent LAD procedure (Reforgiato et al., 2016). Our previous study suggested that ceramide increase occurred at reperfusion and drove inflammatory responses, enhancing myocardial damage (Reforgiato et al., 2016). Therefore, we evaluated the effect of Myriocin in the 24 h following reperfusion, with the aim of investigating if the initial accumulation of this lipid can be responsible for the following remodeling events, specifically in the at-risk area, which is subjected to further damage in the post-infarct phase, and which is the target of post-conditioning. By means of LC-MS/MS analysis, we observed that the level of ceramide was increased by I/R not only in the infarct area, but also in the at-risk area, as compared to sham, and it was reduced by SLN/myr treatment, within the at-risk area (Figure 1C). Since Myriocin inhibits the *de novo* synthesis of ceramide, we also measured the content of its precursor dihydroceramide (Figure 1D). As expected, dihydroceramide accumulated after I/R, and it was significantly reduced by SLN/myr treatment, mirroring ceramide. The amount of the single dihydroceramide and ceramide chains is reported in Supplementary Figures S1, S2, respectively (Supplementary Figures S1, S2). To evaluate the effects of ceramide on pathological remodeling, we analyzed the expression of cardiac specific myosin heavy chains. We observed that Myriocin post-conditioning reverted the infarct-induced altered expression of the myosin chains. In particular, the I/R-induced increased expression of the fetal β-MHC isoform was markedly reduced by SLN/myr treatment (Figure 1E). On the other hand, while I/R injury lowered the expression of the adult α-MHC gene at 24 h in both myriocin treated and non-treated mice, SNL/myr post-conditioning significantly increased the expression of α-MHC at 96 h post-surgery, as compared to untreated animals (Figure 1F). Furthermore, we investigated if the reduction of infarct size could correlate with a reduced development of hypertrophy, another hallmark of adverse remodeling. We measured a significant increment in the HW/BW ratio, representing the extent of left ventricular hypertrophy, in the I/R group, as compared to sham. SLN/myr treatment inhibited this effect (Figure 1G).

### Myriocin Drives a Transcriptional Program Controlling Energy Metabolism

**FIGURE 2:**
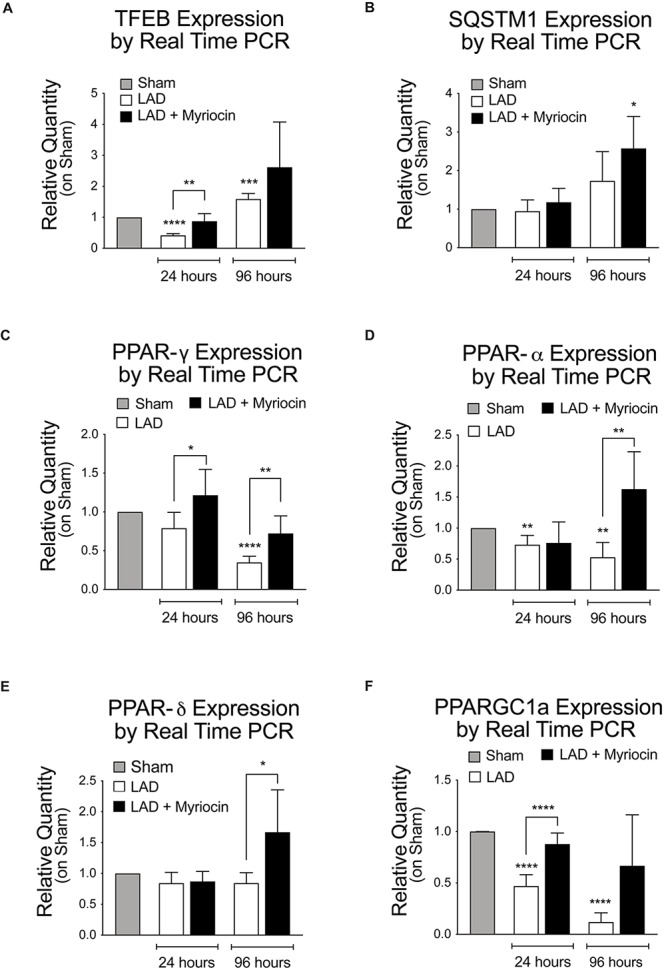
Quantification of TFEB expression levels and gene transcription measurement of PPARs, their activators, and the autophagy-related gene Sqstm1. Quantification of panel **(A)** TFEB RNA levels, along with TFEB target gene transcription SQSTM1 **(B)**. Quantification of PPAR transcription factor family [PPARG **(C)**, PPARA **(D)**, PPARD **(E)**] and of the PPARGC1A co-activator **(F)**. Target gene expressions are measured by qRT-PCR analysis and are expressed as relative quantity on sham group. All measurements are referred to 24 and 96 h after surgery. All data are expressed as mean ± SD. Statistical significance refers to I/R and SLN/myr treated I/R groups as compared to sham animals. The comparison between I/R and SLN/myr treated I/R groups is indicated by the connecting line (^*^p < 0.05; ^∗∗^*p* < 0.01; ^∗∗∗^*p* < 0.001; ^****^p < 0.0001). Sham animals: gray bar; I/R group: white bar; SLN/myr treated I/R group: black bar.

In view of their key role in controlling oxidative stress response and energy metabolism, we investigated the activation of the transcription factor TFEB and of the PPARs nuclear factors family. The expression of TFEB is down-regulated in the immediate post-I/R, but it is significantly enhanced by SLN/myr treatment, as compared to untreated mice (Figure 2A). TFEB transcriptional target SQSTM1 is increasingly up-regulated by Myriocin treatment as well (Figure 2B). In line with literature data, PPARs transcription is down-regulated by I/R stress in myocardium (Abdelrahman et al., 2005; Morrison and Li, 2011). On the contrary, the expression of the key lipid homeostasis regulator PPAR-γ is significantly increased by SLN/myr, as compared to untreated animals, since early time of treatment (Figure 2C). PPAR-α is up-regulated as well, reaching significant increase between Myriocin-treated or untreated mice, at 96 h after post-conditioning (Figure 2D). PPAR-δ expression is only mildly reduced by I/R, but significantly up-regulated by SLN/myr post-conditioning at 96 h (Figure 2E). The expression of PPARs co-activator PGC-1α was investigated as well. PGC-1α transcription was progressively down-regulated after LAD occlusion. As expected, SLN/myr counteracted this inhibition reaching a statistical significance in the increase at 96 h as compared to untreated animals (Figure 2F).

### Ceramide Synthesis Inhibition Modulates the Expression of Genes Involved in Lipid Transport and Mobilization

**FIGURE 3:**
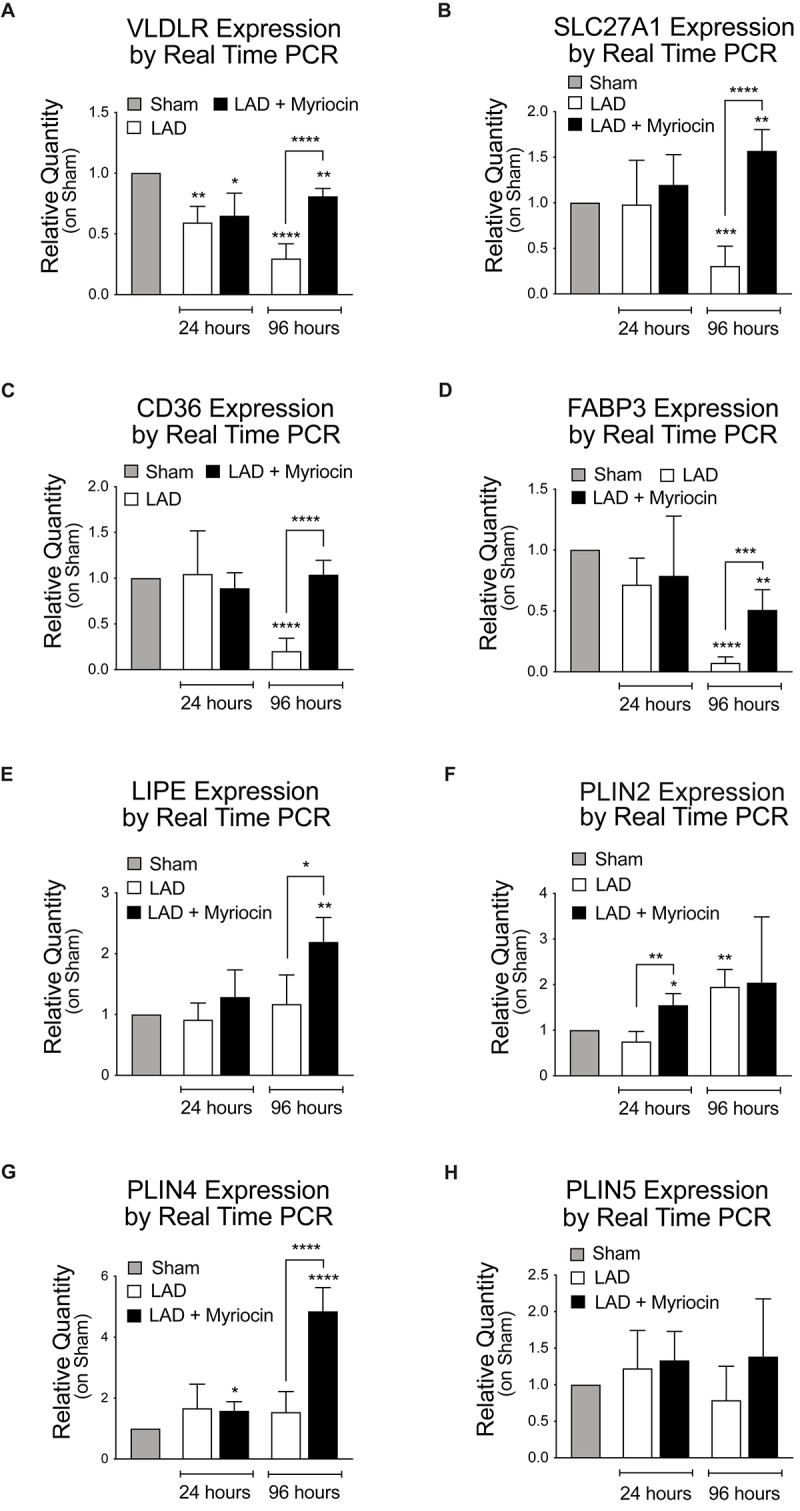
Quantification of the expression levels of lipid transporters and proteins involved in their mobilization. qRT-PCR analysis of the plasma membrane transporters: VLDLR **(A)**, SLCL27A1 **(B)**, CD36 **(C)**, FABP3 **(D)**. qRT-PCR analysis of the FA mobilization enzymes and of the PAT family members: LIPE **(E)**, PLIN2 **(F)**, PLIN4 **(G)** and PLIN5 **(H)**. All data are reported as relative quantity on sham group and are referred to 24 and 96 h after surgery. All data are expressed as mean ± SD. Statistical significance refers to I/R and SLN/myr treated I/R groups as compared to sham animals. The comparison between I/R and SLN/myr treated I/R groups is indicated by the connecting line (^*^p < 0.05; ^∗∗^*p* < 0.01; ^∗∗∗^*p* < 0.001; ^****^p < 0.0001). Sham animals: gray bar; I/R group: white bar; SLN/myr treated I/R group: black bar.

We investigated the expression pattern of FA entry mediators located on the plasma membrane and of their cytosolic transporters: the lipoproteins receptor Vldlr, the lipid transporters Fatp1 (SLC27A1) mediating mainly the uptake of long-chain FA, the CD36 FA translocase, and the cardiac cytosolic transporter FA binding protein (FABP3). All these proteins’ expression was affected by I/R and significantly down-regulated during the following 96 h. SLN/myr treatment recovered the expression of lipid entry/transport regulators and this effect became significant at 96 h post-I/R injury, as compared to Myriocin-untreated mice (Figures 3A–D). Myocardium organizes fat storage by intracellular droplets that take part to metabolism homeostasis. Therefore, we then evaluated the expression of enzymes involved in the mobilization of lipid storage. The hormone-sensitive lipase (HSL) is a cellular lipase interacting with perilipins, which catalyzes the hydrolysis of stored triglycerides in lipid droplets to free FAs (Deng et al., 2006). Interestingly, LIPE gene transcription was significantly increased in SLN/myr treated animals at 96 h post-surgery, as compared to untreated ones (Figure 3E). In parallel, we investigated the effects of SLN/myr on the expression of perilipin genes, belonging to the PAT family of cytoplasmic lipid droplet binding proteins. After I/R injury, all perilipins were up-regulated by myriocin post-conditioning. In particular, PLIN2 expression was significantly increased at 24 h, whereas PLIN4 expression significantly increased after 96 h post-surgery (Figures 3F–H).

### Ceramide Synthesis Inhibition Stimulates FA Oxidation and Energy Production

**FIGURE 4:**
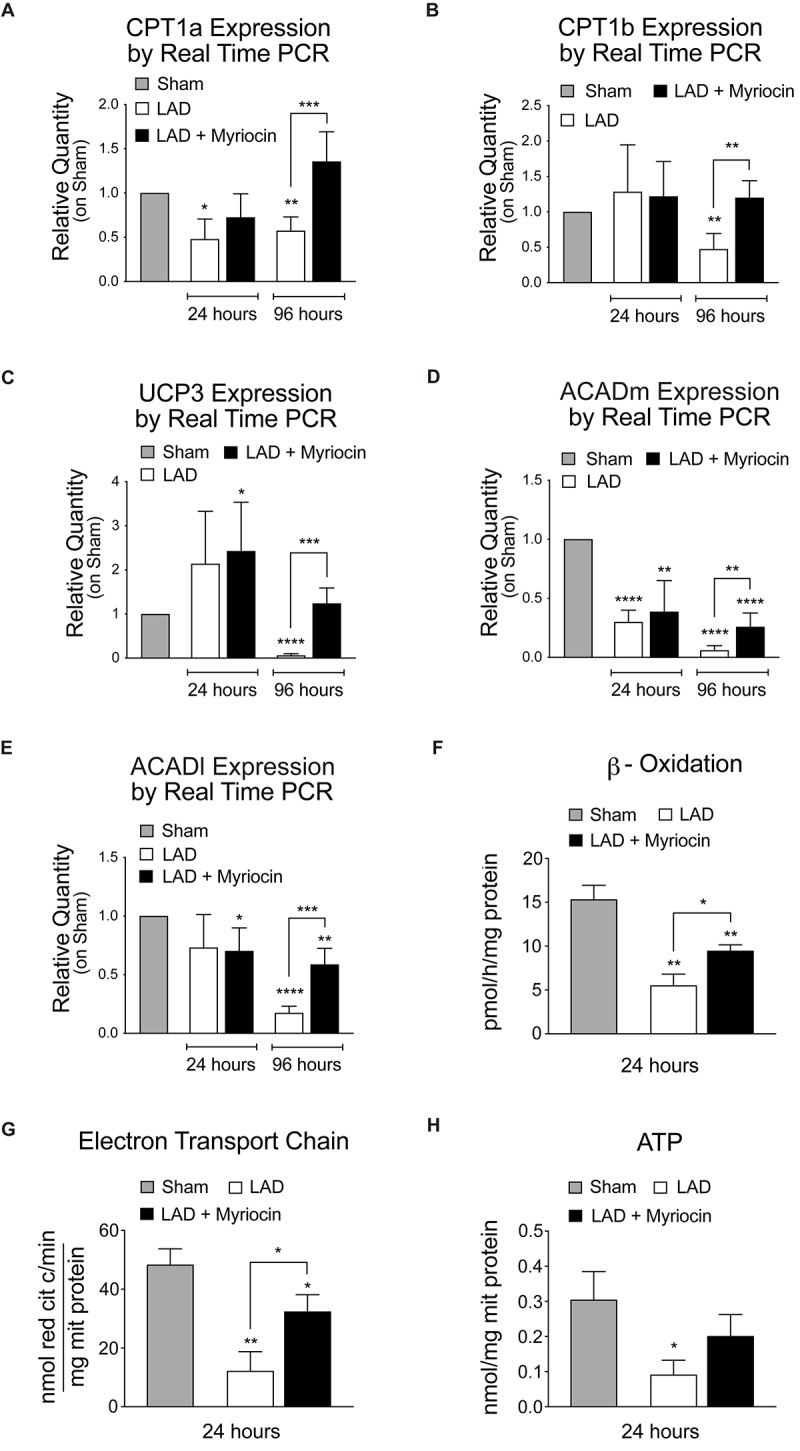
Quantification of mitochondria FA transporters expression, FA oxidation and energy production. qRT-PCR analysis of the mitochondria transporters CPT1a **(A)** and CPT1b **(B)**. qRT-PCR expression analysis of enzymes involved in mitochondrial FAO: UCP3 **(C)**, MCAD **(D)** and LCAD **(E)**. Target gene expressions are measured by qRT-PCR analysis and are expressed as relative quantity on sham group. All measurements are referred to 24 and 96 h after surgery. Analysis of β-oxidation and energy production: β-ox **(F)**, ETC **(G)** and ATP content **(H)**. These measurements are referred to 24 h after surgery. All data are expressed as mean ± SD. Statistical significance refers to I/R and SLN/myr treated I/R groups as compared to sham animals. The comparison between I/R and SLN/myr treated I/R groups is indicated by the connecting line (^*^p < 0.05; ^∗∗^*p* < 0.01; ^∗∗∗^*p* < 0.001; ^****^p < 0.0001). Sham animals: gray bar; I/R group: white bar; SLN/myr treated I/R group: black bar.

In order to understand the fate of lipid mobilization, we investigated the lipid shuttle system associated with the outer mitochondrial membrane, mediated by Cpt1a and Cpt1b proteins, which transport activated FA within the mitochondrial matrix. I/R injury induced a significant reduction of the expression of these transporters at 96 h after surgery in untreated mice. However, SLN/myr post-conditioning counteracted CPT1a and CPT1b expression decrease, inducing a significant increase at 96 h compared to untreated mice (Figures 4A,B). Moreover, we analyzed the expression of the mitochondrial carrier UCP3, which is associated with the survival response to oxidative stress and with FA mitochondrial transport. Results showed a significant down-regulation of UCP3 during the remodeling phase after I/R (96 h), as compared to controls, supporting observations also reported by other authors (Song et al., 2016). On the contrary, SLN/myr post-conditioning significantly enhanced UCP3 expression, as compared to myriocin untreated mice (Figure 4C).

In order to assess whether fat entry and mobilization was finalized at oxidation and energy production, we evaluated the post-conditioning induced transcriptional response of enzymes involved in mitochondrial FAO. MCAD and LCAD, the mitochondrial dehydrogenases that are primarily responsible for β-oxidation of medium and long chains Fas, respectively, were down-regulated by I/R injury, at both time points considered, as compared to sham. Remarkably, Myriocin post-conditioning partially restored, at 96 h, both dehydrogenases expression in treated mice, to levels significantly higher than untreated animals (Figures 4D,E). To support these findings, we measured the FA β-oxidation *ex vivo*, within the at-risk area of SLN/myr treated or untreated myocardium. Interestingly, we observed a significant increase in β-oxidation activity in Myriocin treated animals, as compared to untreated mice (Figure 4F). Since FA oxidation is aimed at energy production, we also evaluated the activity of the mitochondria electrons chain transfer (ETC) and the ATP production. As expected, we observed a significant increase of ETC upon SLN/myr post-conditioning after I/R (Figure 4G), whereas ATP production showed an increasing trend in Myriocin treated animals, as compared to untreated ones (Figure 4H).

## Discussion

We here extended our previous study on Myriocin post-conditioning (Reforgiato et al., 2016) to the remodeling phase occurring in the first days after myocardial I/R. The presented data suggest that modulation of FA oxidation, exerted by Myriocin (SLN/myr), reduces cardiac I/R damage. First, we demonstrated that intraventricular injection of SLN/myr at the beginning of reperfusion significantly affects infarct size up to 4 days from LAD occlusion (Figure 1A). Myriocin inhibits the biosynthesis of sphingolipids, which is enhanced in inflammatory conditions (Caretti et al., 2014; Signorelli et al., 2016) and, specifically, in the myocardium under I/R (Reforgiato et al., 2016). Tissue content of both ceramide and its precursor dihydroceramide was reduced in the at-risk area of SLN/myr-treated myocardium (Figures 1C,D), indicating that ceramide accumulation after I/R was effectively deriving from its *de novo* synthesis and that this mechanism acquired a pathological role. Moreover, Myriocin post-conditioning restrained the extent of the I/R damage, thus preventing the development of cardiac hypertrophy (Figure 1E) and counteracting cardiac myosin heavy chain expression switch (α- and β-MHC), which characterizes adverse remodeling (Figures 1F,G). The metabolism drift, which is associated with reduced lipid consumption for energy production and caused by I/R injury, is sustained by a transcriptional program. Indeed, the expression of genes involved in FA transport and oxidation was already shown to be down-regulated in I/R injury and HF models (Stanley et al., 2005; Amorim et al., 2010). Consequent accumulation of cardiac fat induces lipotoxicity (Schulze, 2009). Lipid mobilization to sustain energy production under stress conditions is a process orchestrated by the transcriptional factor TFEB that promotes lysosomal fat degradation (lipophagy) and the re-activation of their mitochondrial oxidation and ATP production (Settembre and Ballabio, 2014). TFEB is known to activate a set of genes involved in lipid mobilization and oxidation, mostly by the induction of the transcriptional activities of PPARs and their co-activator PGC-1α (Settembre and Ballabio, 2014). Of note, TFEB signaling is suppressed in mouse hearts with advanced cardiac proteinopathy, and it plays a critical role in the cytoprotection in hypoxia/re-oxygenation injury in cultured cardiac myocytes (Pan et al., 2017). Our data show that SLN/myr post-conditioning induces a rapid increase in TFEB mRNA expression (Figure 2A) within the at-risk area, as compared to untreated mice. A TFEB key target is p62-sequestosome, a key component of autophagy vesicles that connect TFEB activation to lipophagy, in support of mitochondrial β-oxidation of FA. Moreover, p62-sequestosome loss is also associated with diabetes and obesity (Angelini et al., 2016). As expected, we found an increased transcription of p62-sequestosome in 96 h SLN/myr-treated myocardial at-risk area (Figure 2B). p62-sequestosome is known to sequester Keap 1 and to release active Nrf2, which potently induce an anti-oxidant response (Jain et al., 2015). We already demonstrated that Nrf2 was induced by SLN/myr post-conditioning in myocardial I/R (Reforgiato et al., 2016) and that Myriocin activated this transcriptional factor to counteract inflammation (Caretti et al., 2016). The activation of PPARs, particularly of PPAR-γ, has been lately proposed as a therapeutic target to limit the extent of myocardial damage after I/R (Abdelrahman et al., 2005; Morrison and Li, 2011). SLN/myr protection from I/R damage was also sustained by the increased transcription of all three isoforms of PPARs and of their co-activator PGC1α (Figures 2C–F). Thus, these results supported the hypothesis that Myriocin enhances lipid catabolism and anti-oxidant response via TFEB and PPARs integrated transcriptional activities, in order to recover cardiac metabolism control in the post-infarct remodeling phase. To confirm this hypothesis, we investigated the expression of specific genes downstream to these transcriptional factors. PPAR-γ was reported to up-regulate the expression of VLDLR (Yagyu et al., 2002; Tao et al., 2010), and such expression is in turn related to the cellular lipid catabolism rate (Yagyu et al., 2002; Tao et al., 2010). PPARs-induced transcription is associated with the activities of key lipid mobilizing enzymes. Specifically, PPAR-γ was reported to induce LIPE Hormone-Sensitive Lipase type E (HSL), the one lipase showing a broader substrates affinity (Deng et al., 2006; de la Rosa Rodriguez and Kersten, 2017). PPARs (mostly α and γ) are reported to promote the expression of genes involved in FA transport (FATP, FAT/CD36, FABP), FA mitochondrial import (CPT-I) and mitochondrial β-oxidation (MCAD, LCAD, VLCAD) (Barger and Kelly, 2000; Nakamura et al., 2014). Among PPARs target genes, there are also the UCP mitochondrial proteins, which mediate FA import and reduce proton gradient and ROS formation (Villarroya et al., 2007). UCP3 is down-regulated in myocardial I/R injury and PPAR-γ activation restores its expression, inducing cardioprotection (Song et al., 2016). Along this line, Myriocin post-conditioning induced an increase in the expression of all the above-reported genes implicated in FA uptake and their mitochondrial transport, demonstrating that the transcriptional program, initiated at 24 h by TFEB and PPAR-γ effectively acted to potentiate cell lipid metabolism in the following hours (Figures 3A–G).

Lipid droplets are considered dynamic stores of metabolic fuel, sustaining cellular FA oxidation and representing a source of inflammatory mediators and gene transcription regulators (Cabodevilla et al., 2013). Lipid excess, especially triglycerides, are stored in lipid droplets. Little lipid droplets accrual normally occurs in the myocardium (Goldberg et al., 2012). Nonetheless, their accumulation associates with many types of stress, including fasting (Suzuki et al., 2001, 2002; Cabodevilla et al., 2013). In isolated cardiomyocytes exposed to anoxia and re-oxygenation, cell death was attenuated in those containing lipid droplets, indicating their cardioprotective effects (Lei et al., 2013). The lipid droplet associated perilipins, PLIN2, 3, 4 and 5, are all expressed in the heart (Paul et al., 2008). PLIN5 is known to be up-regulated by PPAR-α agonists, and it controls lipid homeostasis (Chen et al., 2013) in the myocardium (Paul et al., 2008). Moreover, it was demonstrated to be cardioprotective during myocardial ischemia (Drevinge et al., 2016), and its deficiency significantly increased oxidative stress (Kuramoto et al., 2012; Lei et al., 2013). Of note, patients carrying a minor allele of PLIN5 are at higher risk of cardiovascular morbidity after myocardial ischemic events and characterized by decreased survival (Drevinge et al., 2016). Remarkably, SLN/myr post-conditioning resulted in the up-regulation of all three PLIN isoforms, as compared to untreated animals.

Most importantly, SLN/myr post-conditioning effectively increased FA mitochondria β-oxidation, thus enhancing electron transport and ATP production (Figures 4C–D). These results support that the significant induction of lipid-catabolism, as well as the activation of mitochondrial oxidative processes mediated by Myriocin, could be central in the therapeutic activity of the molecule.

## Conclusion

**FIGURE 5:**
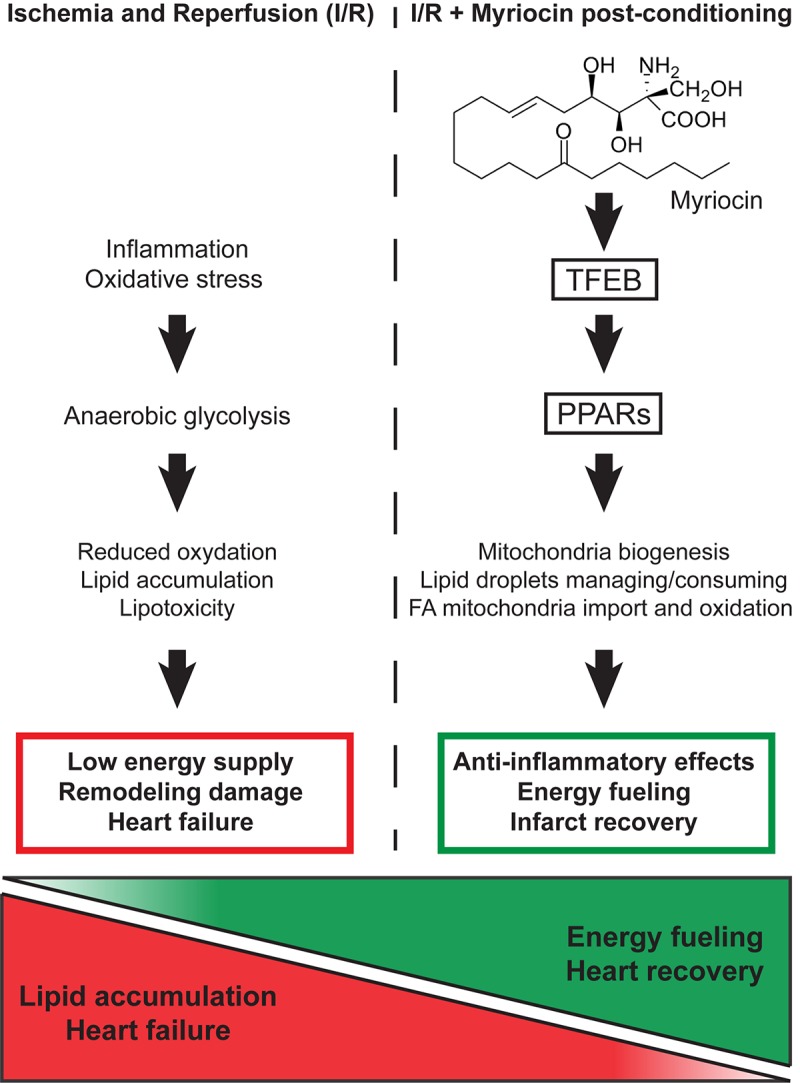
Schematic representation of the molecular mechanism responsible for the Myriocin-induced up-regulation of lipid catabolism and mitochondrial oxidative processes.

The data presented unveiled the molecular mechanisms of Myriocin effects in I/R injury response, which go beyond the simple reduction of ceramide synthesis and extended our previous findings, revealing that Myriocin beneficial effects are not limited to the initial phase of I/R injury, but they can counteract early cardiac remodeling. Along this line, Myriocin post-conditioning activates a transcriptional program that drives the energy metabolism during the I/R injury-induced cardiac remodeling phase. In particular, results support that the inhibition of sphingolipid synthesis induced by the molecule causes a *starvation-like* signal, which eventually modulates the master regulators of lipid consumption, mitochondria formation, and anti-oxidant activities (Figure 5). Indeed, the adverse outcome of myocardial I/R associates with metabolism derangement, lipid accumulation, reduced energy production and impaired contractility. Thus, the capacity of Myriocin to regulate both cardiac inflammation and metabolism after I/R injury support the hypothesis of its use as a possible post-conditioning therapeutic (patent number: US 9925160 B1).

## Data Availability

The datasets generated for this study can be found in the GEO, accession number GSE133913.

## Ethics Statement

Animal studies were conducted in accordance with the Guide for the Care and Use of Laboratory Animals (National Institutes of Health, Publication No. 85–23, revised 1996) and with the ARRIVE guidelines and adhered strictly to the Italian Ministry of Health guidelines for the use and care of experimental animals. Authorization number 361/2017-PR by the Italian Institute of Health (Istituto Superiore della Sanità).

## Author Contributions

RG, LA, and PS conceived the study. FB, MP, MD, AM, AC, and CR performed all the *in vitro* experiments. FB and MP performed all the *in vivo* experiments. FB, MP, LA, and PS designed and analyzed all the experiments. FB, MP, MM, LA, and PS wrote the manuscript. MP, RP, CP, and PS prepared the figures. All authors reviewed the results and approved the final version of the manuscript.

## Conflict of Interest Statement

The authors declare that the research was conducted in the absence of any commercial or financial relationships that could be construed as a potential conflict of interest.

## Supplementary Material

FIGURE S1 LC-MS/MS quantitation of dihydroceramide species in the at-risk area. Dihydroceramide species C-16:0 (^∗∗∗^*p* < 0.001) is increased in I/R sample. Dihydroceramide species C-24:0 (^*^*p* < 0.05) is increased in I/R sample, meanwhile is reduced by SLN/myr treatment vs. I/R group (^∗∗^*p* < 0.01). All data are referred to 24 h after surgery. Data are expressed as mean ± SD. Sham animals: gray bar; I/R group: white bar; SLN/myr treated I/R group: black bar.

FIGURE S2 LC-MS/MS measurement of ceramide species in the at-risk area. Ceramide species C-16:0 (^*^*p* < 0.05) and C-20:0 (^∗∗∗^*p* < 0.001) are increased in I/R sample, meanwhile C-16:0 and C-18:1 are reduced by SLN/myr treatment vs. I/R group (^*^*p* < 0.05). All data are referred to 24 h after surgery. Data are expressed as mean ± SD. Sham animals: gray bar; I/R group: white bar; SLN/myr treated I/R group: black bar.

TABLE S1 Murine primers set to qRT-PCR. Table shows the primers used to perform quantitative real time PCR of target genes.

## Abbreviations

ETC: electron transfer chain
FA: fatty acids
FAO: fatty acids oxidation
LAD: left anterior descending
Myr: Myriocin
SLN: solid lipid nanoparticles.
